# Communications Between Volunteers and Health Researchers during Recruitment and Informed Consent: Qualitative Content Analysis of Email Interactions

**DOI:** 10.2196/jmir.1752

**Published:** 2011-10-13

**Authors:** Anne Townsend, Zubin Amarsi, Catherine L Backman, Susan M Cox, Linda C Li

**Affiliations:** ^1^Arthritis Research Centre of CanadaVancouver, BCCanada; ^2^Occupational Science and Occupational TherapyUniversity of British ColumbiaVancouver, BCCanada; ^3^Maurice Young Centre for Applied EthicsUniversity of British ColumbiaVancouver, BCCanada; ^4^Department of Physical TherapyUniversity of British ColumbiaVancouver, BCCanada

**Keywords:** Emails, recruitment, informed consent, volunteer-researcher interactions, rheumatoid arthritis, qualitative research, motivations to volunteer, help-seeking, self-management

## Abstract

**Background:**

While use of the Internet is increasingly widespread in research, little is known about the role of routine electronic mail (email) correspondence during recruitment and early volunteer–researcher interactions. To gain insight into the standpoint of volunteers we analyzed email communications in an early rheumatoid arthritis qualitative interview study.

**Objectives:**

The objectives of our study were (1) to understand the perspectives and motivations of individuals who volunteered for an interview study about the experiences of early rheumatoid arthritis, and (2) to investigate the role of emails in volunteer–researcher interactions during recruitment.

**Methods:**

Between December 2007 and December 2008 we recruited 38 individuals with early rheumatoid arthritis through rheumatologist and family physician offices, arthritis Internet sites, and the Arthritis Research Centre of Canada for a (face-to-face) qualitative interview study. Interested individuals were invited to contact us via email or telephone. In this paper, we report on email communications from 12 of 29 volunteers who used email as their primary communication mode.

**Results:**

Emails offered insights into the perspective of study volunteers. They provided evidence prospectively about recruitment and informed consent in the context of early rheumatoid arthritis. First, some individuals anticipated that participating would have mutual benefits, for themselves and the research, suggesting a reciprocal quality to volunteering. Second, volunteering for the study was strongly motivated by a need to access health services and was both a help-seeking and self-managing strategy. Third, volunteers expressed ambivalence around participation, such as how far participating would benefit them, versus more general benefits for research. Fourth, practical difficulties of negotiating symptom impact, medical appointments, and research tasks were revealed. We also reflect on how emails documented volunteer–researcher interactions, illustrating typically undocumented researcher work during recruitment.

**Conclusions:**

Emails can be key forms of data. They provide richly contextual prospective records of an underresearched dimension of the research process: routine volunteer–researcher interactions during recruitment. Emails record the context of volunteering, and the motivations and priorities of volunteers. They also highlight the “invisible work” of research workers during what are typically considered to be standard administrative tasks. Further research is needed to fully understand the role of routine emails, what they may reveal about volunteers’ decisions to participate, and their implications for research relationships—for example, whether they have the potential to foster rapport, trust, and understanding between volunteer and researcher, and ultimately shift the power dynamic of the volunteer–researcher relationship.

## Introduction

The Internet is extending research designs [1] and transforming data generation techniques [2] revealing ethical and methodological issues around recruitment [3,4]. A core tool in this transformative process is email, which is routinely used in research [5]. Emails leave an audit trail of volunteer–researcher (or research workers such as research assistants/coordinators) interactions, and record priorities and concerns of volunteers [6]. They also offer insight into the context of a volunteer’s daily life and illness experience, therefore providing a prospective record (rather than a retrospective account) of why or how volunteers decide to participate (or not) in health research.

Email communications may influence volunteer–researcher relationships. Kvale [7] suggests that email interviews potentially alter the power imbalance of participant–researcher interactions, by offering opportunities for a more respectful and symmetrical relationship due to “a shared context of communication” (researcher and participant share a flow of information in their own time and space). This potential shift in the dynamics of research relations may extend to email communications during the recruitment and consent process. For example, email correspondence may extend beyond straightforward recruitment information exchange. Volunteers may disclose negative illness/help-seeking experiences and see volunteering as a way of accessing otherwise unavailable medical advice. Role boundaries can be blurred in these circumstances, and researchers need to be sensitive to the context of participation, but be clear that they cannot offer therapy or medical advice. Also, as emails contain sensitive and personal details, secure and confidential storage is important. All of these factors highlight the skill requirements needed by research workers (eg, research coordinators and assistants) whose role is often seen as purely administrative [8].

Recently, Internet recruitment and email interviews have been the focus of research that has identified ethical, methodological, and practical issues [9]. The potential of routine email correspondence in health research, however, remains unexplored. We addressed this gap by focusing on the content and context of email communications to explore their role in the recruitment and informed consent process and their implications for the volunteer–researcher relationship.

## Methods

### Study Design and Participants

This research formed part of a larger qualitative interview study: Early Rheumatoid Arthritis Help-Seeking Experience (ERAHSE). Rheumatoid arthritis is a chronic musculoskeletal condition and a major cause of disability. The main symptoms are pain, stiffness, joint swelling, and fatigue characterized by exacerbations and remissions [10]. If treatment is delayed, damage can occur in other organs, including the heart and lungs. Onset can be sudden or gradual, and the focus of care is to control the symptoms and limit disease and debility. Timely treatment is crucial to avoid irreversible joint damage, which may lead to permanent disability, increased personal suffering, and medical cost [11].

Individuals who had a diagnosis of rheumatoid arthritis in the previous 12 months, lived in the province of British Columbia (BC), Canada, and were English speakers were eligible. Volunteers were recruited, via information leaflets, through local arthritis clinics and websites of the provincial arthritis organizations. Recruitment documents were also sent to eligible persons by their family practitioner or rheumatologist’s office. The information leaflet invited people to share their experiences of early rheumatoid arthritis in an interview study.

### Data Collection

The information leaflet provided phone numbers and email addresses for 2 members of the research team who would conduct interviews. Out of a sample of 38 participants, 29 used email as their main form of communication. Email was used to confirm eligibility, provide consent forms, discuss queries, and schedule interviews. Typically email communications continued over a period of 1 or 2 weeks. However, in some cases email correspondence extended to several weeks or months due to practical difficulties of scheduling, illness, or life events. The emails were password protected and stored securely on the server of the Arthritis Research Centre of Canada. Factual information (eg, how participants heard about the research) and general comments (eg, length and tone of emails and broad content) were recorded in field notes. Due to the number and content of emails, we recognized they were a rich source of data. We subsequently sought permission to analyze the correspondence of 15 participants, who had engaged in the most email contact overall. The analysis presented here is based on the email communications of the 12 who provided consent (11 females, 1 male). Ethical approval was received from the University of British Columbia Behavioral Research Ethics Board. Volunteers were invited to choose their own pseudonyms, which have been used.

### Data Analysis

The analysis was iterative. A thematic approach informed by a constant comparative method was used. AT and ZA read and coded the emails independently. After discussion and repeated readings of the emails, three initial themes were identified, compared with field notes, and examined for consistency across data types (email, interviews, and field notes). Focusing on emails, further discussion led to agreement on higher-order themes. Constant comparison across data types and scrutiny of all data independently prior to team discussion added rigor to the analysis, contributing to validity of the data-driven claims.

## Results

Volunteer emails varied in number, content, length, and style. Most volunteers noted their diagnostic status and willingness to take part. Some indicated an interest in participating in the ERAHSE study but requested more information about research tasks and the potential risks and benefits of participation. The majority elaborated on their illness situation beyond the eligibility criteria. Several gave richly contextual accounts of their symptoms, medication use, interactions with health professionals, and navigating the health care system [12-15]. Here we focus on four main themes arising from the data: (1) research participation as reciprocity, (2) volunteering as self-managing and help-seeking, (3) ambivalence around participation and informed consent, and (4) practical considerations of participation.

### Research Participation as Reciprocity: Mutual Benefits

Volunteers expressed mutual benefits of participating. In describing their experiences and contributing to the knowledge base, they wanted to help the research initiative and others with early rheumatoid arthritis. At the same time they hoped to benefit from sharing their stories and securing advice or information about illness management. The email below illustrates the perceived twin benefits of participation, for the volunteer and the research endeavor. Nicole volunteers to help the research in the face of frustrating symptoms. She anticipates that sharing her story might help herself and the research, and hopes to gain insight into disease and pain management:

Nicole Hello Anne

My name is Nicole. I am a 33 year-old woman, who was diagnosed with rheumatoid arthritis in about September of last year. I have just, in the last few weeks made contact with the Arthritis Society and received the emailed newsletter, in which I read about the information you are gathering from newly diagnosed patients. I would be happy to talk with someone about my experiences if it would be helpful. I suggest that, as I have been frustrated lately with the disease and with managing pain, that it would help me to talk about it and hopefully gain some insight that I have been missing.

During the subsequent interviews several individuals elaborated on their email disclosures. They expressed hope that their experiences could help our research and future rheumatoid arthritis patients, and assist medical professionals in offering care. At the same time they hoped to benefit through gaining advice and information about available resources. In contrast, some participants reflected on how they made contact solely as a help-seeking strategy, in the face of frustrated attempts to access timely care for worsening symptoms.

### Volunteering as Self-Managing and Help-Seeking

In their initial emails, some volunteers expressed helplessness about their symptoms and frustration at formal health care, making no mention of our study. Some had gained a diagnosis from their family physician and were waiting to see a rheumatologist for effective medications. Several had sought information online and recognized the need for, but were unable to gain, prompt treatment. These volunteers had been induced to contact us in the face of unpredictable, severe, debilitating, or abnormal symptoms and rising anxiety about their situation. The email below illustrates uncertainties around symptoms and concerns about obtaining a timely meeting with a rheumatologist:

Nicolette Regarding my arthritis: a few weeks ago I got inflammation and swelling in both my thumbs. Then 4 weeks ago my finger next to my thumb swelled like a cigar and has stayed that way. Then the joint swelled and became sore and I can see after only a month my finger twisting. Within the last month I have pain in both shoulders as well and in the bone by my wrist as well as my left small toe. IS THIS NORMAL TO COME ON SO FAST? I asked my Dr to send me to a rheumatologist and he told me there was a one-year waiting list to see one. I am in tears and very sad to see my finger twisting right in front of my eyes and I cannot get to see a specialist.

Nicolette described (at interview) the context in which she had emailed us. She had suspected she was in the early stages of rheumatoid arthritis, due to previous knowledge about the disease and an Internet search, which identified the importance of a prompt diagnosis and early treatment. Given this knowledge, and being told by her family physician that there would be a delay of 12 months prior to seeing a rheumatologist, she felt frustrated and sought further information on the Internet. She then found our study and contacted us to talk “to someone” and gain advice.

As illustrated above, some individuals were prompted to volunteer for our study due to frustrated attempts at formal help-seeking. They viewed research participation as a way of accessing much needed support and advice in their quest for prompt treatment. Emails raised questions such as “What should I do?” and included comments such as “I might learn something [if I take part]”, indicating volunteer need (for support in and access to help-seeking) and perceived benefits of participation. This posed potential ethical problems for free and informed consent.

### Ambivalence Around Participation

Email correspondence revealed questions about informed consent and offered some insight into the decision-making process. Rain made contact hoping for help in navigating the health care system and was in “two minds” about participating. In his initial email, he described his frustration with the health care he was receiving and did not refer to the research study. Subsequently, he asked a series of questions about accessing care while considering whether to take part. Rain emphasized that his primary motivation for making contact was his hope for a speedy rheumatologist referral, which was difficult to obtain in his rural community. His ambivalence about participating was apparent in his questions about the informed consent document, as he asked explicitly about the risks and benefits:

Rain Hi Anne

Thank you for your concern, I was originally looking for a study for a cure or therapy...

I would like to get some understanding about the section – Risks and Potential Benefits–in the consent form it mentions “It is possible that some topics discussed may raise new and sometimes difficult issues...” What sort of issues should one be concerned about? As mentioned above, I’m looking for a cure to get rid of my daily pain. An interview may help you, but I’m still suffering.

Thank you.

Rain elaborated in his interview that he “went on the Internet to look for some support” [15, p 23]. The email record offers a glimpse of the prospective decision-making process from the volunteer perspective, rather than through hypothetical or retrospective concerns around consent—for example, when eliciting responses during an interview. This is a vivid illustration that informed consent is a 2-way flow that extends beyond ensuring volunteers have received the consent form prior to interview, to review at the time of interview. It also highlights the need to be flexible regarding communication formats. In this example the volunteer agreed to a phone conversation regarding his concerns and any potential risks, burdens, or benefits of participating for him, compared with potential benefits for others and the research more generally.

### Practical Considerations of Participation

After receiving the recruitment documents, several volunteers focused on practical aspects of participation. They reported busy lives characterized by symptom management and hospital appointments, and gave insight into the research experience as they negotiated a convenient time and place for interview. The emails also offered volunteers the opportunity to set out the parameters of participation. In the correspondence below, Teresa notes her preferred location and three suitable times for the interview, asking the researcher to let her know what “works best”:

Teresa Hello,

I looked over the attachments and everything’s ok. After consideration I think it would be too long a day for me to add the interview into a (hospital) appointment. The [occupational therapist] appointments tend to go on for 1 ½ hours or more. My rheumatoid arthritis is very active right now and I’m easily fatigued. I would prefer a home interview, which would be more relaxed and give better insight into the impact of my rheumatoid arthritis. Possible dates are Monday April 7 1pm, Tuesday April 8 10 am or Friday April 11 10 am.... Let me know what works best.

This email records a daily life, compromised by symptoms and treatment, and adds context to the data generated at interview. It records the potential burdens and costs of the research task for this participant in real time (ie, the efforts taken in order to participate in a research interview study). It also highlights the importance of a convenient time and place for collecting data, for the comfort of the participant and the quality of the data gained.

## Discussion

### Principal Results

The email communications offered insight into the perspectives of volunteers in our study. They generated prospective data on motivations to take part, recruitment, and informed consent. First, we found there was a reciprocal element to participating. Some volunteers felt they could be of help to research and at the same time hoped that participating would be of help to them. Second, others were prompted to volunteer due to their acute need for information in the face of troublesome symptoms and frustrations with the health care system. For these individuals volunteering was solely a self-management or help-seeking strategy. Third, some ambivalence was illustrated when deciding to participate, regarding the difference between potential benefits for the volunteer and benefits for the research in general. Fourth, practical difficulties of participation arose—for example, scheduling an interview in the context of a daily life organized around symptom containment and medical appointments. Finally, the emails also revealed rarely discussed dimensions of the volunteer–researcher interactions and the invisible work of researchers. Overall, our findings contribute new knowledge to the scant information on the ethics involved in email communications [16,17].

### Context of Volunteering: Experience of Early Illness and Help-Seeking

In common with those in other studies, our volunteers hoped to gain health benefits [18]. People who are in the early stages of a chronic illness may well experience uncertainty and anxiety about their condition [12,14,15]. Such feelings may be exacerbated when people are not provided with a firm diagnosis or prompt treatment [12]. In this context, people who feel that they are unheard in the health care system and are aware that they require timely treatment may be inclined to volunteer for research about their condition. Our recruitment materials described an interview study and an interest in personal experiences. This may well suggest an outlet for a personal illness story to be heard and promise hope of advice or support in a patient’s quest for a speedy diagnosis or effective treatment. More research is needed to identify how far people volunteer for research to access information or advice as part of their self-management and help-seeking strategies.

### Recruitment, Informed Consent, and Volunteer–Researcher Interactions

The emails provided prospective records of aspects of recruitment, consent, and volunteer–researcher interactions, and as such generated data on an underinvestigated dimension of the research process. Emails facilitate a 2-way flow of information exchange, in a “shared context of communication” [7], and have the potential to contribute to a more collaborative health research relationship in the era of the informed epatient [19]. The volunteers in our study had the opportunity to interact in their own time and space at their own convenience, rather than in a face-to-face situation or via the more immediate and (possibly) intrusive telephone. This may have shifted the balance of control and offered the possibility of an active volunteer [6] participating in a more meaningful and involved recruitment and informed consent process. For example, individuals may have been more inclined to enter into a prolonged dialogue about participation and to broach “sensitive” issues when deciding whether to take part. The volunteer emails in our research recorded reasons for taking part (Nicole) and doubts, reflections, and questions about benefits and risk (Rain). Volunteers may also share emotional stories and frustrations (eg, Nicolette) or, on a practical basis, take the initiative in terms of when and where the interview should take place (eg, Teresa). In future research projects it would be instructive to ask the participants their views on the role of email communications with researchers and research workers during recruitment.

### Potential for Rapport Building Through Email Communications

The opportunities for building more collaborative and dynamic relationships in electronic health care [20] applies equally to the qualitative health research process. “The emergent nature of many qualitative studies makes the achievement of rapport with participants and feelings of interpersonal trust crucial to the generation of questions considered important or interesting by both parties” [21 p3]. For this community of volunteers with newly diagnosed rheumatoid arthritis, emails offered a way to engage in dialogue at an anxious and frustrating time, and provided opportunities to foster trust and rapport (eg, one participant requested to be interviewed by the researcher with whom she had been emailing) [14]. This potential benefit mirrors what Childress [22] describes as “proximal benefit:” both the participant and researcher can potentially gain from building an appropriate but respectful relationship during the research process.

### The Invisible Work of Research Workers

The volume and content of emails surprised us, motivated this analysis, and highlighted the undocumented or invisible work of researchers and research coordinators [8]. Given the context of some volunteers’ illness stories and navigating the health care system, such communications may create expectations regarding the nature of the response. Although we needed to be sensitive to volunteers’ circumstances, and emails offered opportunities to build trust and rapport, we also needed to negotiate the boundaries between acting as “therapist” and acting as “sensitive researcher.” This necessitated time for prompt, careful, and informative correspondence as we attempted to achieve a careful balance between (objective) pragmatism and (subjective) empathy in the context of multiple tasks and deadlines. Also, given the context of some volunteers’ illness stories and their reported frustrations at “not being heard,” research workers need perhaps to consider how volunteers are informed that they do not fulfill study criteria.

### Practical Obstacles to Participation

The emails pinpointed the practical costs of participation for the volunteers. The real-time communications suggested that for these participants, the burdens of taking part in interviews held some practical obstacles—for example, the potential to aggravate symptoms such as fatigue. More research is needed to assess whether such considerations are relevant beyond this dataset and for volunteer patients in all types of health research.

### Limitations

Our findings are limited in scope. As in all qualitative research we do not claim to make generalizations, but to gain a more in-depth understanding of social phenomena. We drew on a small number of emails from study volunteers with newly diagnosed rheumatoid arthritis, who perhaps were particularly keen to participate due to their help-seeking and illness experiences. We highlight that this analysis of the email communication included 1 male and 11 females and could not undertake a gender comparison. In the future, it would be preferable to study a gender-balanced sample. It would also be beneficial to include all of the emails from volunteers rather than a selection. We suggest, however, that the findings can be usefully explored in a range of research settings and designs. Below we offer some observations regarding practice, future research, and educational initiatives.

### Practice

Standard operating procedures created at study inception are one way to ensure all research staff approach communications in a thoughtful and consistent way. These procedures should include mechanisms for secure handling and storage of emails, and it should remain clear that study participants be informed of the risks to privacy when using email and that they may prefer (and should be offered) alternative means of communication.

### Suggestions for Research

To better understand the role of email use in health research, we need more evidence on emails and how this form of communication may influence recruitment, informed consent, and volunteer–researcher relationships, as well as the skill set needed by research workers. Exploratory research questions could include “What is the nature and extent of routine email communications in different research populations and what are the potential challenges and benefits?”

### Educational Aspects

On the basis of our findings, we cannot recommend extensive educational interventions. However, we suggest that educational workshops, which focus on emerging ethical issues in the use of new technologies, could include sessions on email communications. Workshops could engage those involved in research, such as ethicists, health research participants, and researchers, to identify and reflect on emerging issues. Comparisons could be made between phone and email communications in terms of ease of use, content, form and language, and interpersonal relations. Topics could include issues around using emails as data and securing consent to do so.

### Conclusion

We are unaware of other studies that have been based on the analysis of volunteer recruitment emails. The emails tracked part of the decision-making process in real time, recording volunteers’ hopes, concerns, and practical contingencies in the context of their illness experience. Because emails can be a rich, prospective data source, researchers may wish to include them as data, which has implications for consent. Although this is a small sample, from which we cannot make general statements, volunteers in other contexts may see health research participation as a way to access care, information, and advice. Research workers should be aware of this during the recruitment and informed consent process.

Emails are not only a technological development but also a reformulation of recruitment and informed consent offering the potential for increased dialogue during routine communications in health research. A key implication of this study is how email communications revealed the invisible work of research workers during recruitment and informed consent. Using the emails as data improved our understanding of the decision-making process, the context in which people volunteered for our study, and the practical obstacles involved. There was a suggestion that emails fostered opportunities for meaningful and thoughtful dialogue over time, but more research is needed to investigate this and perhaps their capacity to shift the dynamics from a traditional to a more symmetrical relationship, as well as a more considered informed consent process.

## Abbreviations

ERAHSE: Early Rheumatoid Arthritis Help-Seeking Experience
